# Oral Health Status of Chinese Children With Autism Spectrum Disorders

**DOI:** 10.3389/fpsyt.2020.00398

**Published:** 2020-05-05

**Authors:** Yanan Qiao, Han Shi, Hui Wang, Mingbang Wang, Fengshan Chen

**Affiliations:** ^1^Department of Orthodontics, School and Hospital of Stomatology, Tongji University, Shanghai Engineering Research Center of Tooth Restoration and Regeneration, Shanghai, China; ^2^Department of Pediatrics, Xiamen Branch of Children's Hospital of Fudan University (Xiamen Children's Hospital), Xiamen, China; ^3^Shanghai Key Laboratory of Birth Defects, Division of Neonatology, Xiamen Branch of Children's Hospital of Fudan University (Xiamen Children's Hospital), Children's Hospital of Fudan University, National Center for Children's Health, Shanghai, China

**Keywords:** autism spectrum disorders, oral health, children, questionnaire, China, halitosis

## Abstract

**Objectives:**

To assess and compare the oral health status of children with and without autism spectrum disorders (ASD) in China.

**Methods:**

This study recruited 144 children with ASD and 228 unrelated children with typical development (TD) aged 3–16 years from China. Data were collected using parent-reported questionnaires. Oral problems (oral symptoms and habits), oral health measures (oral hygiene practice and dental care experience), and the impact on the child's quality of life (based on a modified version of the Parental-Caregiver Perception Questionnaire) were assessed and compared between the two groups.

**Results:**

Children with ASD had worse oral health status than children with TD. Oral symptoms were more prevalent in the ASD group, especially halitosis (p < 0.001), food impaction (p < 0.001), and oral lesions (p < 0.001), than the TD group. The rate of damaging oral habits, including mouth breathing (p < 0.001) and object biting (p < 0.05), was also high in the ASD group. Compared with the TD group, more children with ASD did not brush their teeth independently and frequently (p < 0.001), had difficulty accessing dental care (p < 0.01), and reported unpleasant dental experiences (p < 0.001). The presence of ASD was associated with decreased oral health-related quality of life (p < 0.001) in these children and their families.

**Conclusion:**

Oral problems such as halitosis and bad oral habits are more prevalent among children with ASD. These children also lack oral hygiene practice and dental visits. This situation negatively impacts their quality of life, and must be brought to the attention of their treating dentists.

## Introduction

Autism spectrum disorders (ASD) affect one in 59 children aged 8 years in the United States, with four to five males receiving a diagnosis of ASD for every female who is diagnosed (1). A large-scale study estimated the prevalence of ASD in China was approximately 1% (2), though no official figures are currently available. This suggests there is a high possibility that dentists will encounter children with autism during their careers. ASD is a life-long heterogeneous psychiatric disorder that is characterized by impaired social behavior, communication, and language and concomitant restriction in interests and activities (3). ASD may potentially influence the oral health of affected children, and present challenges for dental care (4, 5).

Children with ASD are widely reported to have poorer oral hygiene (6–9) and worse periodontal conditions (6, 7, 9, 10) than healthy children. Damaging oral habits such as bruxism (excessive grinding/clenching of teeth), tongue thrusting, and lip biting are also common among children with ASD (6, 8, 9). Moreover, children and adolescents with ASD are regarded as a high-risk group for dental caries (6, 7, 11–13), and are prone to self-injury and dental trauma—even self-extraction of teeth (9, 14). Studies performed in American, European, and Middle Eastern countries have reported conflicting findings regarding these issues (7, 9–11, 15–20). However, the risk for oral problems among Chinese children with ASD remains unexplored. In addition, existing evidence of an increased prevalence of oral disease among children with ASD is contradictory and inadequate (21, 22). Most previous oral health data for children with ASD were derived from relatively small and often clinical samples, which have an overt bias compared with population/community-based samples.

The present study aimed to fill this gap by conducting a population-based oral health survey to explore oral problems, oral health behaviors, and the impact of oral status on quality of life among Chinese children with ASD compared with children that exhibited typical development (TD). Clarifying these issues will support dental professionals to better respond to the special needs of this vulnerable group of children.

## Materials and Methods

### Participants

We enrolled 144 children with ASD (mean age 8.10 ± 3.31 years, 84% boys) and 228 age-matched children with TD (mean age 8.01 ± 3.07 years, 53% boys) in this study (**Table 1**). Children with ASD were recruited through rehabilitation centers and non-governmental organizations for patients with autism from three areas in China (Shenzhen, Xiamen, and Shanghai). We explained the purpose of the study to the principals of the centers and invited them to participate. The inclusion criteria for children with ASD were: boys or girls younger than 16 years of age with a diagnosis of ASD from Shanghai Mental Health Center, Xiamen Children's Hospital, or Shenzhen Children's Medical Center based on guidelines set out in the Diagnostic and Statistical Manual of Mental Disorders, Fifth Edition.

**Table 1:** Demographic profile of children with autism spectrum disorder and those with typical development.

Characteristics	ASD group (n=144)		TD group (n=228)		p value
	n	%	n	%	
Patient's age (ys)^†^					0.66
3-6	53	36.55%	76	33.33%	
7-11	67	46.53%	123	53.95%	
12-16	24	16.67%	29	12.72%	
Patient's gender^‡^					<0.001***
Male	121	84.03%	121	53.07%	
Female	23	15.97%	107	46.92%	
Parent's education level, n (%)					
Postgraduate	14	9.72%			
College/University	58	40.28%			
Junior college	37	25.69%			
High school	26	18.06%			
Middle school	8	5.56%			
Elementary school	1	0.69%			
Family annual income, n (%)					
Upper	10	6.94%			
Upper middle	29	20.14%			
Lower middle	48	33.33%			
Upper lower	36	25.00%			
Lower	21	14.58%			

ASD, autism spectrum disorder; TD, typical development; ^†^Calculated continuously using the t-test; ^‡^Calculated using chi-square test. ^***^Differences significant at p < 0.001.

The exclusion criteria were: diagnosis of other mental illness (e.g., attention-deficit hyperactivity disorder, obsessive compulsive disorder, depression), other neurodevelopmental disorder, genetic metabolic disease, or severe neurological disease; history of brain injury; or history of other major physical illness. Children in the TD group were recruited from the communities in the same districts through an online forum. Inclusion criteria for the TD group were: healthy, without mental illness, and age-matched with the ASD group. The exclusion criteria were the same as for the ASD group. This study was approved by the Ethics Committee of the School and Hospital of Stomatology, Tongji University. Parents of all participating children signed an informed consent form before the study started. Efforts were made to protect participants' privacy. All study-related information was stored securely on personal computers.

### Questionnaire

The questionnaire included two sections. The first section covered basic information (e.g., the child's age and gender, and family socioeconomic status), bad oral habits (eight habits: nail/pencil biting, finger/nipple sucking, tongue thrusting, mouth breathing, lip biting, unilateral chewing, drooling, and bruxism), oral hygiene measures (e.g., type and frequency of tooth brushing), and previous dental experiences (e.g., frequency and reason for visiting a dentist). The response options for the items covering the oral habits were “positive” and “negative,” whereas the other questions in this section were answered according to the specific situation (as shown in **Tables 1** and **3**). An “I don't know” response was also permitted, with these answers excluded from the analysis.

The second section comprised a modified version of the Parental-Caregiver Perception Questionnaire (P-CPQ) and the Family Impact Scale (FIS), which aimed to assess parents' perceptions of their children's oral health-related quality of life and its effect on the family (23). The P-CPQ has been validated in English (23) and Chinese (24). Given that the Early Childhood Oral Health Impact Scale and the P-CPQ have similar properties but differ in the applicable scope for age (25), we combined these questionnaires (mainly based on the P-CPQ for 8–10-year-olds). We also presented the FIS as part of this scale (26). Therefore, the modified P-CPQ developed for this study had 27 items on five subscales: oral symptoms, functional limitations, emotional wellbeing, social wellbeing, and the FIS. We used the FIS to roughly evaluate parental emotions and family finances. All items were scored on a 5-point Likert-type scale: never=0, once or twice=1, sometimes=2, often=3, and every day/almost every day=4. The mean scores for all domains and the overall P-CPQ were obtained, with high scores denoting oral conditions had a negative impact on the children's quality of life (**Table 4**). The questionnaires for the ASD group were completed by the children's parents (mothers) when they accompanied their children to the rehabilitation centers. Questionnaires for the TD group were completed online.

### Statistical Analysis

Student's t-tests were used to determine the significance of the differences in continuous variables between the two groups. Continuous variables were presented as means and standard deviations, and the outcomes were checked for a normal distribution using a Kolmogorov–Smirnov test. Variables that were not normally distributed were analyzed using a non-parametric Mann–Whitney U test. Categorical data were compared using chi-square tests. Fisher's exact test was used if the observed frequency in a single cell was below 5. We calculated the Cronbach's alpha to show the reliability of the modified P-CPQ, and used the Kaiser-Meyer-Olkin test to show its validity. Multiple linear regression analysis was used to assess the effect of sociodemographic factors, with P-CPQ items as dependent variables. A p-value <0.05 was considered statistically significant. All statistical analyses were performed using SPSS version 21.0 (IBM corp. Armonk, NY, USA).

## Results

**Figure 1:**
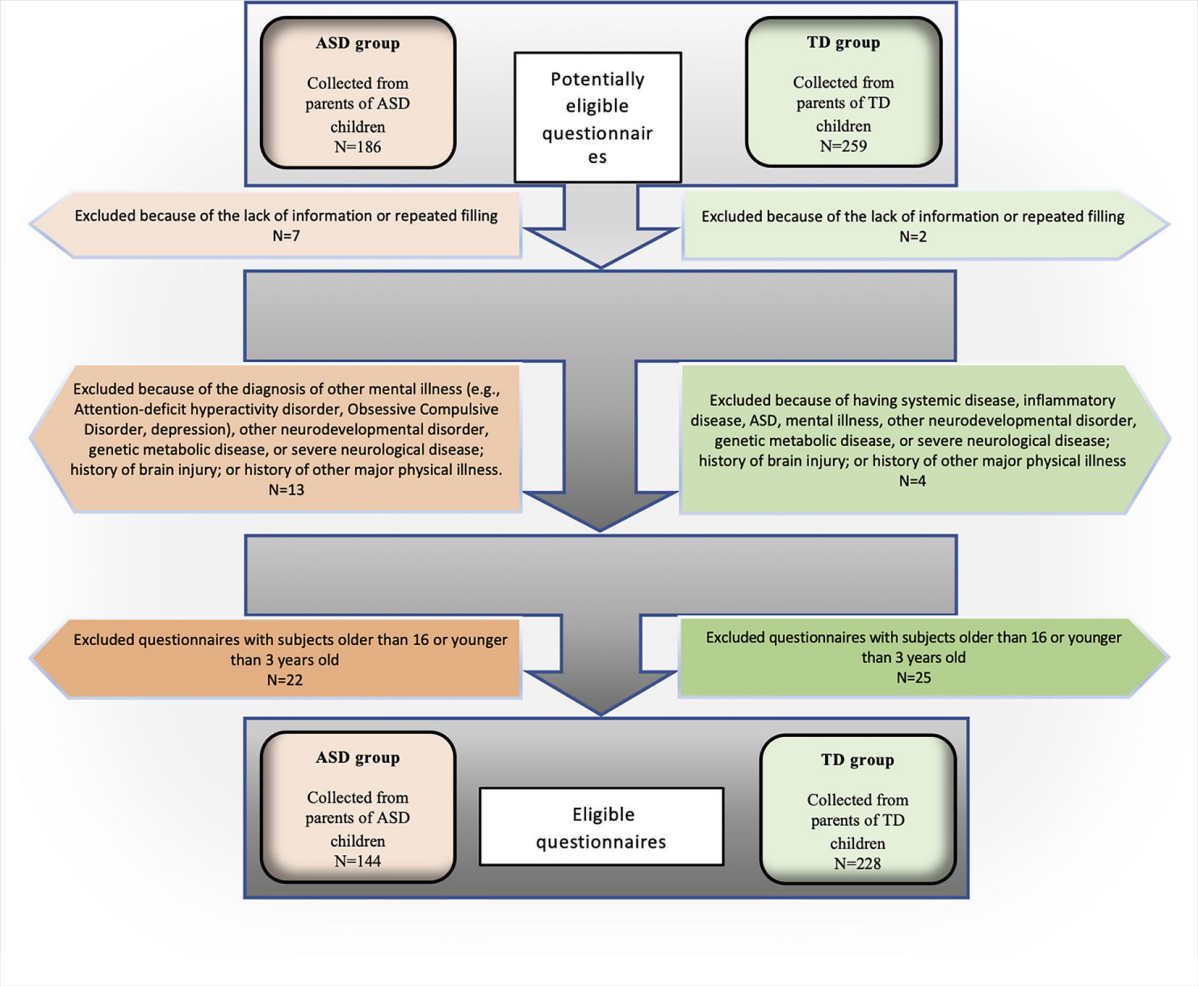
Flow diagram of included and excluded questionnaires.

We collected 372 eligible questionnaires from a pool of 445 questionnaires completed by parents of children with ASD and TD. **Figure 1** presents a flow diagram of the screening and identification process. The ASD group comprised 144 children aged 3–16 years (mean age 8.10 years) (**Table 1**). The majority of the children with ASD were boys (84%), with a male to female ratio of 5:1. The TD group included 228 children aged 3–16 years (mean age 8.02 years), with a male-to-female ratio of 1:1 (**Table 1**). In both groups, approximately half of the children were in the 7–11-year age group (47% of the cases and 54% of the controls). **Table 1** presents participants' demographic characteristics.

We investigated the frequencies of oral problems in all participants (e.g., halitosis, dental caries, bleeding gums, food impaction, oral pain, and oral lesions) along with various oral habits. We considered that a particular symptom that sometimes, often, or frequently occurred as positive, and one that never or rarely occurred as negative (25). Although most participants generally experienced oral symptoms to some degree, children with ASD experienced significantly more symptoms (99.2%; 118/119) than those in the TD group (94.1%, 206/219; p < 0.001) (**Table 2**). Specifically, 95.6% of the ASD group experienced halitosis, with this noticeably and significantly more common than in the TD group (69.9%; p < 0.001). The rate of food impaction was the second most prevalent symptom, and was also higher in the ASD group (79.4%) than in the TD group (69.5%; p < 0.001). In addition, oral lesions, pain, and bleeding gums were more prevalent in children with ASD than those with TD (oral lesions: 62.5 vs. 17.4%, p < 0.001; pain: 47.9 vs. 30.3%, p < 0.01; bleeding gums: 41.9 vs. 30.9%, p < 0.05). These findings indicated there was an association between ASD and poor oral health. However, parents of children in the ASD group reported similar rate of tooth decay in their children compared with the TD group (**Table 2**). Overall, the five most prevalent oral symptoms in children with ASD were halitosis (95.6%), food impaction (79.4%), oral lesions (62.5%), dental caries (56.4%), and oral pain (47.9%).

**Table 2:** Comparison of oral problems between the autism spectrum disorder group and the typical development group.

Characteristics	ASD group (n=144)		TD group (n=228)		p value
	n	%	n	%	
Oral symptoms					
Halitosis^†^	130/136	95.56%	154/222	69.87%	<0.001***
Food impaction^†^	104/131	79.39%	133/219	69.47%	<0.001***
Oral lesions^†^	85/136	62.50%	39/224	17.41%	<0.001***
Dental caries^†^	79/140	56.43%	135/240	56.25%	0.97
Oral pains^†^	57/119	47.90%	69/228	30.26%	<0.01**
Bleeding gums^†^	57/136	41.91%	68/220	30.91%	<0.05*
None^†^	1/119	0.84%	13/219	5.94%	<0.001***
Oral habits					
Mouth breathing^†^	49/144	34.03%	38/228	16.67%	<0.001***
Nail/Pencil biting^†^	45/144	31.25%	47/228	20.61%	<0.05*
Drooling^†^	25/144	17.36%	22/228	9.65%	<0.05*
Bruxism^†^	24/144	16.67%	51/228	22.37%	0.18
Finger/Nipple sucking^†^	22/144	15.28%	23/228	10.09%	0.14
Tongue thrusting^†^	19/144	13.19%	19/228	8.33%	0.13
Lip biting^†^	14/144	9.72%	10/228	4.34%	<0.05*
Unilateral chewing^†^	11/144	7.64%	14/228	6.14%	0.57
None^†^	18/144	12.50%	86/228	37.72%	<0.001***

ASD, autism spectrum disorder; TD, typical development. ^†^Calculated using the chi-square test. ^*^Differences significant at p < 0.05; ^**^Differences significant at p < 0.01; ^***^Differences significant at p < 0.001.

In terms of bad oral habits (**Table 2**), mouth breathing was markedly more common in children with ASD (34%) than in children with TD (16.7%), with the difference being highly significant (p < 0.001). Biting hard objects (i.e., nails, pencils, and toys.) was also more prevalent in the ASD group (31.3%) than in the TD group (20.6%; p < 0.05). Moreover, children in the ASD group showed a much higher rate of unconsciously drooling and lip biting compared with the TD group (drooling: 17.4 vs. 9.7%, p < 0.05; lip biting: 9.7 vs. 4.3%, p < 0.05). The prevalence of other bad oral habits, such as finger sucking, bruxism, tongue thrusting, and unilateral chewing showed no significant differences between children with and without ASD. Only 12.5% of parents of the ASD group reported no bad oral habits, compared with 37.7% of those in the TD group (p < 0.001). The five most prevalent bad oral habits in children with ASD were mouth breathing (34%), biting hard objects (31.3%), drooling (17.4%), bruxism (16.7%), and finger sucking (15.3%).

The results for items covering oral hygiene and dental experiences are shown in **Table 3**. There were significant differences in the frequency of tooth brushing (p < 0.001) and independent tooth brushing (p < 0.001) between the ASD and TD groups. In the ASD group, 57/144 (39.6%) of children brushed their teeth twice per day and 58/144 (40.3%) brushed once per day. In contrast, 139/228 (61%) of children with TD brushed twice and 85/228 (37.3%) brushed once per day. Out of those who were brushing, only 23/142 (16.2%) of children with ASD brushed their teeth autonomously, versus 166/221 (75.1%) of controls (p < 0.001). A majority of the former underwent fully assisted brushing or required the help of their parents/caregivers. Toothpaste is another important consideration when comparing routine tooth brushing; the use of fluoride toothpaste was slightly lower in the ASD group (49.5%) compared to the TD group (53.1%).

**Table 3:** Comparison of oral health behaviors between children with autism spectrum disorder and those with typical development.

Characteristics		ASD group (n=144)		TD group (n=228)		p value
		n	%	n	%	
Oral hygiene practices	Type of brushing^§^					<0.001^***^
	Autonomous	23/142	16.20%	166/221	75.11%	
	Assisted	48/142	33.80%	11/221	4.98%	
	Both	66/142	46.48%	49/221	22.17%	
	Brushing frequency^§^					<0.001^***^
	Never	13/144	9.03%	1/228	0.44%	
	Sometimes	16/144	11.11%	1/228	0.44%	
	Once a day	58/144	40.28%	85/228	37.28%	
	Twice a day	57/144	39.58%	139/228	60.96%	
	Three times or more	0/144	0.00%	2/228	0.88%	
	Use of flouride toothpaste^‡^					0.32
	Yes	54/109	49.54%	94/177	53.11%	
	No	55/109	50.46%	83/177	46.89%	
Recent dental experience	Recent frequency to see a dentist^§^					0.004^**^
	Once in 1 month	11/135	8.15%	28/223	12.56%	
	Once in 3 months	12/135	8.89%	20/223	8.97%	
	Once in 6 months	16/135	11.76%	47/223	21.08%	
	Once in 1 year or more	26/135	19.26%	67/223	30.04%	
	Never	70/135	51.85%	61/223	27.35%	
	The previous dental experience^§^					<0.001^***^
	Very pleasant	5/110	4.55%	78/169	46.15%	
	Pleasant	24/110	21.82%	56/169	33.14%	
	Normal	24/110	21.82%	19/169	11.24%	
	Unpleasant	41/110	37.27%	11/169	6.51%	
	Very unpleasant	16/110	14.55%	5/169	2.96%	
	Reason for the dental visit					
	Routine examination^‡^	18/108	16.67%	41/216	18.98%	0.61
	Dental caries^‡^	32/108	29.63%	67/216	31.02%	0.80
	Bleeding gums^‡^	4/108	3.70%	4/216	1.85%	0.31
	Surgical treatment^‡^	16/108	14.81%	36/216	16.67%	0.67
	Other reason^‡^	21/108	19.44%	43/216	19.91%	0.92

ASD, autism spectrum disorder; TD, typical development. ^‡^Calculated using chi-square test; ^§^Calculated using Mann-Whitney U test. ^*^Differences significant at p < 0.05; ^**^Differences significant at p < 0.01; ^***^Differences significant at p < 0.001.

The analysis of previous dental experiences showed significant differences in the recent frequency of visiting a dentist between children with and without ASD (p=0.004, **Table 3**). Nearly half of those with ASD had never been to a dentist, and out of the other half who had visited, 51.8% had encountered an unpleasant (or very unpleasant) experience. This rate was significantly higher than among children exhibiting TD (9.5%, p < 0.001). The top three reasons for visiting a dentist for both the ASD and TD groups were (in order): dental caries, routine examination, and surgical treatment.

To evaluate parents' perceptions of their child's oral health-related quality of life, the modified P-CPQ were involved (**Table 4**). The Cronbach's alpha for the modified P-CPQ was 0.88, and the Kaiser-Meyer-Olkin measure of sampling adequacy was 0.82 (p < 0.001, Bartlett's test of sphericity), indicating good internal consistency reliability and construct validity. The mean scores for oral symptoms, functional limitations, emotional wellbeing, social wellbeing, and FIS in the ASD group were 8.42, 9.66, 11.06, 5.42, and 6.47, respectively. The corresponding mean scores in the TD group were 6.04, 5.00, 4.82, 2.68, and 2.59, respectively. There were statistically significant differences between the two groups in all domains as well as in overall P-CPQ scores (all p < 0.001) (**Table 4**). This study showed that children with ASD had alarmingly higher scores than those with TD. Given that high scores denoted a negative impact of oral conditions on the children's quality of life, this finding indicated that oral problems had a major impact on children with ASD and their families. To minimize the bias from the wide age range, we also calculated scores for the subgroup of children aged 7–11 years, which represents the key period for tooth replacement (**Table 4**). The mean scores for the oral symptoms and the overall P-CPQ in this subgroup were higher compared for those calculated for all participants, and the differences were significant.

**Table 4:** Impact of oral problems for children with autism spectrum disorder and typical development showing the modified Parental-Caregiver Perception Questionnaire scores.

Characteristics	No. of items	All participants			7-11 years old participants		
		Mean score (± SD)		p value	Mean score (± SD)		p value
		ASD group (n=144)	TD group (n=228)		ASD group (n=67)	TD group (n=123)	
Modified P-CPQ^†^	27	41.12 ± 18.02	21.05 ± 12.13	<0.001^***^	41.91 ± 12.02	21.06 ± 1.41	<0.001^***^
Oral symptoms^†^	5	8.42 ± 2.12	6.04 ± 2.73	<0.001^***^	9.13 ± 3.70	6.34 ± 2.82	<0.001^***^
Functional limitation^†^	5	9.66 ± 2.83	5.00 ± 3.01	<0.001^***^	9.25 ± 4.09	4.25 ± 2.71	<0.001^***^
Emotional well-being^†^	8	11.06 ± 3.54	4.82 ± 5.07	<0.001^***^	10.57 ± 6.68	4.72 ± 4.84	<0.001^***^
Social well-being^†^	5	5.42 ± 4.05	2.68 ± 2.84	<0.001^***^	6.39 ± 4.48	2.66 ± 2.69	<0.001^***^
FIS^†^	4	6.47 ± 3.80	2.59 ± 2.65	<0.001^***^	6.77 ± 3.08	2.38 ± 2.46	<0.001^***^

ASD, autism spectrum disorder; TD, typical development; SD, standard deviation; P-CPQ, Parental-Caregiver Perception Questionnaire; FIS, Family Impact Scale. ^†^Calculated using the t-test. ^***^Differences significant at p < 0.001.

Finally, a multiple linear regression model was used to evaluate the effect of different sociodemographic factors on parental perceptions of children's quality of life and the impact on their families in the ASD group (**Table 5**). The results showed that age was the only factor that had a significant and positive impact on modified P-CPQ scores. This suggested that as children with ASD grow older, they tended to have higher P-CPQ scores and oral conditions had a worse impact on their quality of life. The children's gender, annual family income, and parents' graduation level did not show significant associations.

**Table 5:** Multiple linear regression with modified P-CPQ scores as dependent variable to demonstrate the sociodemographic variables on parental perceptions of oral health-related quality of life in the autism spectrum disorder group.

Modified P-CPQ Model	Unstandardized coefficients		Standardized coefficient	t	Sig.
	B	Sth. error	β		
(constant)	36.126	7.797		4.633	.000
Gender	-7.001	3.971	-.143	-1.763	.080
Age	1.407	.443	.259	3.177	.002^**^
Education level	1.103	1.507	.067	.732	.465
Annual income	-1.842	1.459	-.116	-1.263	.209

ASD, autism spectrum disorder; P-CPQ, Parental-Caregiver Perception Questionnaire. ^**^Differences significant at p < 0.01.

## Discussion

### Oral Problems Experienced by Children With and Without ASD

This study aimed to investigate oral health problems associated with ASD in a Chinese population. We found that 99.2% of children with ASD suffered from (at least one) oral comorbidities, including halitosis, food impaction, oral lesions, and oral pain, with rates of these symptoms significantly higher than in the TD group (**Table 2**). Although several prior studies have explored the oral health status of participants with ASD, none of these studies considered halitosis. We found that 95.6% of parents of children with ASD indicated their child suffered from halitosis, significantly higher than those without ASD. Halitosis is an offensive odor that originates from the oral cavity as a result of microbial metabolism on the tongue dorsum or in the saliva (27). Our previous study put forward a direct correlation between ASD and oral microbial dysbiosis (28, 29), which may directly lead to the oral malodor of these children. Various oral, systemic, and psychological diseases can be sources of halitosis (30). Intra-oral sources includes food impaction, oral lesions, poor oral hygiene, and poor gingival and periodontal condition (27, 30), all of which were observed to be prevalent in children with ASD in this study (**Table 2**). Impacted and residual food debris can be degraded by bacteria and produce volatile sulfur compounds that contribute to bad breath (27, 31). In addition, many studies have detected high rate of gastrointestinal comorbidities in patients with ASD, ranging from 9–70%, such as gastrointestinal disease, digestive disorders, and constipation (32). These factors may be extra-oral reasons for halitosis in children with ASD (27). Further studies investigating the occurrence and severity of halitosis in patients with ASD are warranted.

Oral pain and oral lesions are also regarded as manifestations of oral and systemic disease (33). Consistent with previous studies (9, 22, 34–36), we found that children with ASD had significantly higher rates of oral pain and lesions (**Table 2**), which were linked to toothache, oral ulceration, and dental and mucosal traumatisms. This may be because children with ASD are prone to repeated self-injury behaviors, and lack caution in situations of risk (7, 35). Although many studies have indicated higher amounts of dental caries in children with ASD (7, 12, 15, 19), we found a similar prevalence of caries (according to parental report) in the ASD and TD groups. No or little difference of caries risk has also been reported in studies using dental examinations conducted in Western countries and Hong Kong, which might have been due to the similar plaque pH and saliva buffering capacity in children with and without ASD (18, 20, 22, 37).

Various oral habits were also observed in most children with ASD in this study (**Table 2**). Recent studies reported that the prevalence of oral habits reached up to 94.3% among those with ASD aged 4–23 years and 87.3% among preschool children with ASD (38, 39). In this study, the most common oral habit was mouth breathing followed by object biting. We found the prevalence of mouth breathing in children with ASD was 34%, which was similar to previous findings of approximately thirty percent (38, 40, 41). A recent study also reported a significantly higher rate of disordered breathing in an autistic population compared with controls (41). This improper breathing pattern has an adverse effect on the tongue, jaw, and head posture during a child's development phase, and may be a predisposing factor for differences in facial growth (42). Mouth breathers were reported to show a significant increase in halitosis, probably because the oral cavity becomes dry due to the evaporation of saliva when the mouth remains open, thereby causing bad breath (43). Dental and medical specialists should recognize that normalizing the breathing pattern plays an important role in the facial development of growing individuals with ASD. Early diagnosis and treatment should be performed if possible, such as wearing an oral appliance and excising swollen adenoids.

In terms of other damaging oral habits (**Table 2**), we found that children with ASD were prone to bite foreign objects such as nails and pencils, which may explain the high rate of self-inflicted oral lesions (7, 44). Object biting, lip biting, and pica eating have been reported to be more commonly seen in autistic children in other studies (45, 46). A previous study also reported that unconscious drooling of saliva was more common in individuals with ASD compared with controls (9), which was consistent with our finding. Poor masticatory function, swallowing difficulties, and pouching of food in children with ASD have also been reported (9, 47). These problems may be attributable to decreased oral muscle tone and coordination (48).

### Oral Health Behaviors and Dental History of Children With ASD and TD

The majority of studies unequivocally pointed to inadequate oral hygiene in children and adults with ASD compared with unaffected individuals (21, 35, 45, 47), although many individuals with ASD receive regular assistance with tooth brushing (4, 14, 35, 45). We also found that children with ASD were incapable of brushing their teeth independently (**Table 3**). This might be because of their limited manual dexterity and sensory issues (4, 49), which present challenges to the parents/caregivers when providing oral care for children with ASD. We observed a significantly low frequency of tooth brushing in children with ASD, and 9% of these children did not brush at all. It is necessary to raise parents' awareness of the importance of routine dental hygiene practice, and motivate them to guide their children to brush twice a day in an effective manner so that adequate oral hygiene can be achieved. It has been reported that the brushing effectiveness of children with ASD can be improved *via* visual pedagogy and video-modeling brushing intervention (50, 51).

We observed that 21% of children in the TD group saw a dentist once every 1–3 months (**Table 3**), which may reflect routine dental checkups of school children by local dental hospitals. However, more than half of the children with ASD in our study had never been to a dentist (**Table 3**). A recent study conducted in Pakistan reported that 81% of autistic children had never been to a dentist (19), which was a higher rate than found in this study. The main barrier to dental visits may lie in the child's behavior, cost of treatment, and lack of insurance. Of those children with ASD who had received dental care, more than half had encountered an unpleasant or very unpleasant experience (**Table 3**). Several prior studies also found children with ASD usually exhibited uncooperative behavior during dental procedures (9, 18, 19, 36).Children with ASD are prone to anxiety and temper tantrums when exposed to an unfamiliar dental setting and become non-compliant and uncooperative for the dental procedure (14). Other factors that may make oral health treatment unpleasant for children with ASD include: limited verbal communication, heightened sensory perception, resistance to receiving dental care, possible avoidance of social contact, and urgent need for dental treatment, along with the inexperience with ASD on part of the dental clinician (12, 19, 36, 52). Therefore, dental teams may need to predict children's behavior and apply management techniques to better deal with children with ASD (53).

### Impact of Oral Problems on Quality of Life for Children With ASD

Oral health-related quality of life, reflected by oral symptoms, functional limitations, emotional wellbeing, social wellbeing, parental emotions, and family finances, was significantly lower in children with ASD than TD in this study (**Table 4**). This indicates that the daily life of children with ASD and their families was disturbed on account of their oral problems. Previous studies also reported that childhood autism resulted in a reduced oral and overall health-related quality of life for both the affected children and their families (54, 55). Our regression analysis (**Table 5**) showed that the child's age played a significant role in the model. This suggested that quality of life is likely to be more negatively impacted as a child grows older. It is known that oral diseases tend to worsen with age and seriously impair the individual's quality of life (56). Therefore, as a child grows, it becomes more important to take good care of their oral health. Proper oral healthcare, especially prevention and early treatment, may be potentially important for children with and without ASD to enhance their quality of life.

### Limitations

Although this study benefited from its relatively large sample size, some limitations should be noted. First, this study covered a broad age range (3–16 years), which might have caused bias, despite allowing the explore of oral manifestations at different developmental stages. Heterogeneity in the oral health status with age was previously reported, with more caries in older children (20, 47). Oral habits were also reported to be more prevalent in preschool children (57). Therefore, further subgroup analysis by age is recommended. Second, the gender distribution in the TD group was equal whereas the male to female ratio in the ASD group was 5:1. Although consistent with national statistics for ASD of 4–5:1 (1), this gender difference might have impacted our study results. Another limitation was that no data were collected about parental income and educational level in the TD group, thereby a comparative analysis of the impact of these sociodemographic factors between groups and whether the significant role of age seen in the ASD group was also present in the TD group was not possible. In addition, as we did not capture and organize data by the severity of ASD, meaning we could not explore associations between the oral health status and type of autistic symptoms. This limited the generalizability of the results to the ASD population. Finally, using parental-reported data was also a limitation in this study, as bias might have been introduced, especially when questions were related to oral symptoms where social desirability and non-professional estimation may impact reporting. Thus, clinical oral examination is necessary to confirm the prevalence of halitosis, caries, and oral lesions, and clarify the severity of these problems in patients with ASD; this area merits further research. In addition, given that admitting bad oral habits and poor hygiene practices may cause shame, acknowledgement of these habits or practices may be greater in face-to-face interviews than in a relatively anonymous parental-report situation.

## Conclusion

Despite these limitations, this work demonstrates that there were differences in oral health status between children with and without ASD in China. Children with ASD showed a high prevalence of halitosis, oral lesions, mouth breathing, and drooling. The main reasons for the high rate of oral problems in children with ASD may be lack of tooth brushing and dental visits. Our study suggests that ASD results in reduced oral health-related quality of life for both affected children and their families, especially for older children. Parents should be motivated and guide their children to brush their teeth more frequently. Dental teams should also be aware of this situation, and make efforts to develop novel strategies to provide proper oral care to this special-needs population.

## Data Availability Statement

The datasets generated for this study are available on request to the corresponding authors.

## Ethics Statement

The studies involving human participants were reviewed and approved by the Ethics Committee of the School and Hospital of Stomatology, Tongji University. Written informed consent to participate in this study was provided by the participants' legal guardian/next of kin.

## Author Contributions

MW, YQ, and FC designed the study. YQ, HS, MW, and HW distributed the questionnaires. YQ analyzed the data and wrote the manuscript. FC, MW, and HS revised the manuscript. All authors have read and approved the manuscript.

## Conflict of Interest

The authors declare that the research was conducted in the absence of any commercial or financial relationships that could be construed as a potential conflict of interest.
